# Novel cryoballoon to isolate pulmonary veins in patients with paroxysmal atrial fibrillation: long-term outcomes in a multicentre clinical study

**DOI:** 10.1007/s10840-022-01200-5

**Published:** 2022-04-12

**Authors:** Andrew Martin, Marina Fowler, Toni Breskovic, Alexandre Ouss, Lukas Dekker, Sing-Chien Yap, Rohit Bhagwandien, Elizabeth M. Albrecht, Nele Cielen, Elizabeth Richards, Binh C. Tran, Nigel Lever, Ante Anic

**Affiliations:** 1 Green Lane Cardiovascular Services, Auckland Mail Centre, Auckland City Hospital, Private Bag 92 189, Auckland, 1142 New Zealand; 2 University Hospital Center Split, Split, Croatia; 3 Catharina Hospital, Eindhoven, Netherlands; 4 Erasmus MC, Rotterdam, Netherlands; 5 Boston Scientific Corp., Electrophysiology, St. Paul, MN USA

**Keywords:** Novel cryoablation system, Atrial fibrillation, Pulmonary vein isolation

## Abstract

**Background:**

Recently, a novel cryoballoon ablation catheter has demonstrated acute safety and efficacy in *de novo* pulmonary vein isolation (PVI) procedures in patients with paroxysmal atrial fibrillation (PAF). However, there are limited studies demonstrating the long-term efficacy. The aim of this study was to evaluate the long-term safety and efficacy of this novel cryoballoon in treating PAF.

**Methods:**

This was a non-randomized, prospective, multicentre study enrolling 58 consecutive patients. Cryoablation was delivered for 180 s if time to isolation was ≤ 60 s. Otherwise a 240-s cryoablation was performed. One centre performed pre- and post-ablation high-density mapping (*n* = 9) to characterize lesion formation. After a 3-month blanking period, recurrence was defined as having any documented, symptomatic episode(s) of AF or atrial tachycardia. All patients were followed for 1 year.

**Results:**

Acute PVI was achieved in 230 of 231 pulmonary veins (99.6%) with 5.3 ± 1.6 cryoablations per patient (1.3 ± 0.7 cryoablations per vein). Forty-three (77%) patients remained arrhythmia-free at 1-year follow-up. Four patients (6.9%) experienced phrenic nerve injury (3 resolved during the index procedure; 1 resolved at 6 months). One serious adverse device event was reported: femoral arterial embolism event occurring 2 weeks post-index procedure. For patients who underwent high-density mapping, cryoablation was antral with 50% of the posterior wall ablated.

**Conclusions:**

Initial multicentre clinical experience with a novel cryoballoon has demonstrated safety and efficacy of PVI in patients with PAF. Ablation with this cryoballoon provides a wide, antral lesion set with significant debulking of the posterior wall of the left atrium.

## Introduction

Pulmonary vein isolation (PVI) ablation is a well-established guideline recommended treatment for atrial fibrillation (AF) [1]. There are currently numerous approved energy sources and technologies for achieving PVI. These include point-by-point radiofrequency energy, phased radiofrequency, cryoballoon ablation, and endoscopic laser balloon [2–4]. Cryoballoon ablation has become a common *de novo* strategy due to more predictable procedure times, high efficacy, favourable safety profile, and “single shot” approach to creating antral PVI [2, 5]. Despite these highly appealing characteristics, this system does have some drawbacks. One of these is the small increase in balloon size during the initial freeze cycle [5]; this can disrupt the position of the balloon, in turn reducing ablation efficacy.

Recently, a novel cryoballoon system (POLARx, Boston Scientific) has become commercially available. Unlike the existing commercially available system (Arctic Front, Medtronic), the POLARx cryoballoon maintains uniform pressure and size throughout the freeze cycle. However, given this cryoballoon is more compliant, there have been concerns raised regarding the stability of balloon placement, with potential for more ostial lesion formation, and resultant issues for procedure safety and subsequent long-term outcomes [6].

Initial data on this POLARx system have demonstrated successful acute PVI and safe procedural outcomes [7–13]. To date, one study has completed 12-month follow-up in a small patient subset with 71% of patients remaining arrhythmia-free [10]. However, more clinical data are required, including safety profile, lesion characterization, and long-term efficacy. The aim of this study was to demonstrate the acute performance, characterize the lesion set achieved, and evaluate the long-term safety and efficacy in treating paroxysmal AF using the POLARx cryoballoon system.

## Methods

This was a non-randomized, single arm, prospective, multicentre study. Patients were enrolled as part of the continued access protocol (NCT03723070), which includes 58 patients enrolled after the initial first-in-human cohort previously reported [10]. The study was conducted in accordance with the Declaration of Helsinki and was approved by the ethics committees and local regulatory agencies at all participating sites. Informed consent was obtained from all patients prior to enrollment.

Patients were enrolled from four centres in Europe (Croatia, the Netherlands) and New Zealand. Fifty-eight patients consecutive with paroxysmal AF undergoing *de novo* PVI were enrolled in this study. Patients included in this study had previously failed to achieve arrhythmia control while on class I or III antiarrhythmic drugs. The exclusion criteria for this study were described previously [10] and include AF lasting longer than 7 days, a history of cerebral infarct, transient ischaemic attack or systemic embolism, and more than 4 electrical cardioversions in the year prior to the enrollment. During pre-operative imaging, patients with PV diameter greater than 30 mm or a common left PV were excluded from this study.

### Study design

The objective of this study was to demonstrate acute and chronic safety and performance of the novel cryoablation system. Study endpoints were described previously [10]. The primary safety endpoint was freedom from device- or procedure-related serious adverse events at 12-month post-procedure. The primary efficacy endpoint was acute procedural success, with PVI confirmed via exit and entrance block testing. Secondary endpoints included all procedure- and device-related adverse events, treatment success defined as the proportion of subjects free from symptomatic atrial arrhythmias at 12 months post-procedure (including fibrillation, flutter, and tachycardia), and cryoballoon procedural characteristics (procedure duration, fluoroscopy time, cryoablations per vein, and single application success rates).

### Cryoablation procedure

The use of anticoagulation and oral antiarrhythmic medications leading up to the procedure was at the discretion of the operator following standard of care for the institution. Prior to the procedure, patients underwent pre-procedural TEE, CT, or intracardiac echocardiography (ICE) during the procedure to confirm the absence of left atrial (LA) appendage thrombus. Intravenous heparin was administered with repeat doses administered as necessary to achieve an activated clotting time (ACT) greater than 300 s prior to the transseptal puncture and then an ACT of greater than 350 s prior to ablation.

Patients underwent PVI using the POLARx cryoballoon system. The sheath (POLARSHEATH) was introduced through the femoral vein. A single or double transseptal puncture was performed following institutional standard of care. Prior to cryoballoon ablation, baseline mapping of the PVs was performed using a commercially available circular mapping catheter (at the discretion of the treating team) followed by the circular mapping catheter (POLARMAP). After mapping, the cryoballoon catheter — short tip (5 mm) or long tip (12 mm) — was introduced and navigated to the PVs. Prior to each application, occlusion of the PV was verified using contrast venography graded on a scale of 1 to 4. Grade 4 indicating complete occlusion with no visible leak.

The duration of cryoablation delivered was based on an algorithm using the time to effective PVI [14]. Cryoablation applications were 180 s in duration if the time to isolation (TTI) was 60 s or less. Otherwise, a 240-s cryoablation application was performed. A minimum application time of 120 s was required for each PV. No bonus freeze applications were performed. Oesophageal temperature monitoring was used during the procedure and if temperature fell below 25 °C, the cryoablation was immediately stopped. In addition, right phrenic nerve pacing was performed during cryoablation of the right PVs to reduce risk of phrenic nerve injury. Phrenic nerve capture was monitored using a movement sensor (Diaphragm Movement Sensor, Boston Scientific) and with conventional means which included palpation or visual monitoring via X-ray or ICE. If there was a loss of phrenic nerve capture, the cryoablation was immediately stopped.

A 30-min waiting period was performed based on operator’s standard of care but was not mandatory. Acute PVI was confirmed with entrance and exit block testing using a commercially available circular mapping catheter. If isolation was not achieved or reconnection occurred, repeat cryoablation applications were performed using the same dosing protocol with a freeze time of 180 s for TTI less than or equal to 60 s. Otherwise, a 240-s cryoablation application was performed.

### Lesion set characterization

Patients enrolled after the initial first-in-human cohort of nine patients from a single centre (Auckland, New Zealand) had an electroanatomical map created using an Advisor Circular Mapping Catheter (Abbott Medical) and high-density 3D mapping system (EnSite Precision, Abbott Medical). The inclusion criteria for these patients were the same as for others enrolled in the trial. Following PVI, a bipolar voltage map was obtained to determine the extent of pulmonary vein and posterior wall isolation. These maps were merged with a rendering of the LA created using the CT angiogram which all patients had prior to the procedure. The border of the PVI was denoted by the complete absence of electrograms, with a voltage cut-off < 0.2 mV. Voltages > 0.5 mV were considered to represent unablated tissue. A volumetric technique using a trapezoidal model was used to quantify the extent of vein and posterior wall electrical isolation in accordance with previous studies [15].

### Post-procedure

Post-ablation, patients had in-office follow-up visit at discharge (usually the day after the procedure) and at 1-, 3-, 6-, and 12-month follow-up. During the follow-up visits, a physical exam was performed along with a 12-lead ECG. In addition, 24-h Holter monitor was recorded at 3-, 6-, and 12-month follow-up.

### Statistics

Continuous variables were reported as mean ± standard deviation. Categorical variables were summarized as count and percentage. Twelve-month recurrence was calculated both as a binomial rate with exact 95% confidence intervals and using Kaplan–Meier methodology. Short and long tip catheter 12-month binomial recurrence rates were compared using a chi-square test. Statistically significant differences across PVs (left superior, LSPV; left inferior, LIPV; right superior, RSPV; and right inferior, RIPV) were identified using repeated measures analysis of variance, accounting for within subject correlation (SAS Version 9.4, SAS Institute software company). A *p*-value less than 0.05 was considered significant and a post hoc paired *t*-test analysis, applying a Bonferroni correction, was performed if significance was reached.

## Results

Fifty-eight patients with paroxysmal AF underwent PVI with the novel cryoballoon catheter system, performed at 4 centres. Baseline demographics are displayed in Table 1.

**Table 1:** Patient characteristics

Characteristic	Result
Age (years)	59 ± 10
Male (*N* (%))	37 (63.8)
Height (cm)	179 ± 11
Weight (kg)	86 ± 14
Comorbidities (*N* (%))	
Diabetes mellitus	4 (6.9)
Hypertension	17 (29.3)
Congestive heart failure	0 (0)
Arrhythmia history (*N* (%))	
Atrial flutter	12 (20.7)
Atrial tachycardia	3 (5.2)
Medication at time of procedure (*N* (%))	
Class 1 antiarrhythmic	13 (22.4)
Class 3 antiarrhythmic	9 (15.5)
CHA_2_DS_2_-VASc score (*N* (%))	
0	21 (36.2)
1	16 (27.6)
2	16 (27.6)
3	5 (8.6)

### Procedural data and cryoballoon performance

The mean procedural time for PVI ablation was 108 ± 26 min across all 58 cases. A 30-mi waiting period was not mandatory but was performed in 24 out of 58 cases. The mean fluoroscopy time was 14 ± 9 min.

All PVI procedures were performed using only the POLARx cryoballoon. Acutely, 231/232 (99.6%) PVs were successfully isolated, with one PV without documented exit and entrance block testing. Thirty-four patients were treated with the long tip cryoballoon catheter, 22 were treated with the short tip catheter, and in two patients both the short and long tip cryoballoon catheters were used. In total, 306 cryoablations were performed, resulting in a mean of 5.3 ± 1.6 cryoablations per patient (1.3 ± 0.7 cryoablations per vein). Fifteen cryoablations were terminated prior to 120 s (2 cryoablations stopped at 119 s).

All cryoablations achieved a grade 3 (8%) or grade 4 (92%) occlusion, with similar performance across individual PVs (Table 2). Electrical isolation of the PV with a single cryoablation was achieved in 175 veins (76%), and 23 patients (40%) had successful PVI with only 4 cryoablations (1 per vein). The cryoablations per vein and single ablation success rates were similar across PVs and are shown in Table 3. Further, no differences in procedural characteristics or cryoballoon performance were seen between cases performed with the short or long tip cryoballoon catheters.

**Table 2:** Procedural characteristics

Procedural characteristics	All (*N* = 58)	With 30-min waiting period (*N* = 24)	Without 30-min waiting period (*N* = 34)
Procedure time (min)	107.6 ± 26.1	121.9 ± 22.8	97.5 ± 23.7
Fluoroscopy time (min)	14.1 ± 9.0	12.2 ± 7.7	15.5 ± 9.7
LA dwell time (min)	98.3 ± 25.7	111.5 ± 23.2	87.3 ± 22.7
Cryoablation time (min)	17.0 ± 5.4	17.7 ± 6.0	16.5 ± 5.0

Data displayed as mean + / − standard deviation

**Table 3:** Cryoballoon performance data by pulmonary vein

	LSPV* (*N* = 58)	LIPV (*N* = 57)	RSPV (*N* = 58)	RIPV (*N* = 58)	All PVs (*N* = 231)
Acute isolation success rate	100%	100%	100%	98.3%	99.6%
# of cryoablation application	1.3 ± 0.6	1.3 ± 0.7	1.4 ± 0.8	1.3 ± 0.6	1.3 ± 0.7
# of veins treated with a single cryoablation application	44 (76%)	45 (79%)	42 (72%)	44 (76%)	175 (76%)
Nadir temperature (°C)	− 54.2 ± 5.4	− 49.9 ± 4.5	− 56.6 ± 5.4	− 52.6 ± 5.3	− 53.3 ± 5.7
Occlusion grade					
Grade 3	1 (2%)	7 (12%)	4 (7%)	6 (10%)	18 (8%)
Grade 4	57 (98%)	50 (88%)	54 (93%)	52 (90%)	213 (92%)

*LIPV*, left inferior pulmonary vein; *LSPV*, left superior pulmonary vein; *RIPV*, right inferior pulmonary vein; *RSPV*, right superior pulmonary vein

^*^One PV recorded as LSPV was a left common PV

Of the 24 patients that had a 30-min waiting period, three patients (four PVs) had at least one additional ablation performed due to acute PV reconnection. All patients were then re-treated successfully with the POLARx cryoballoon.

### Lesion characterization

**Fig. 1:**
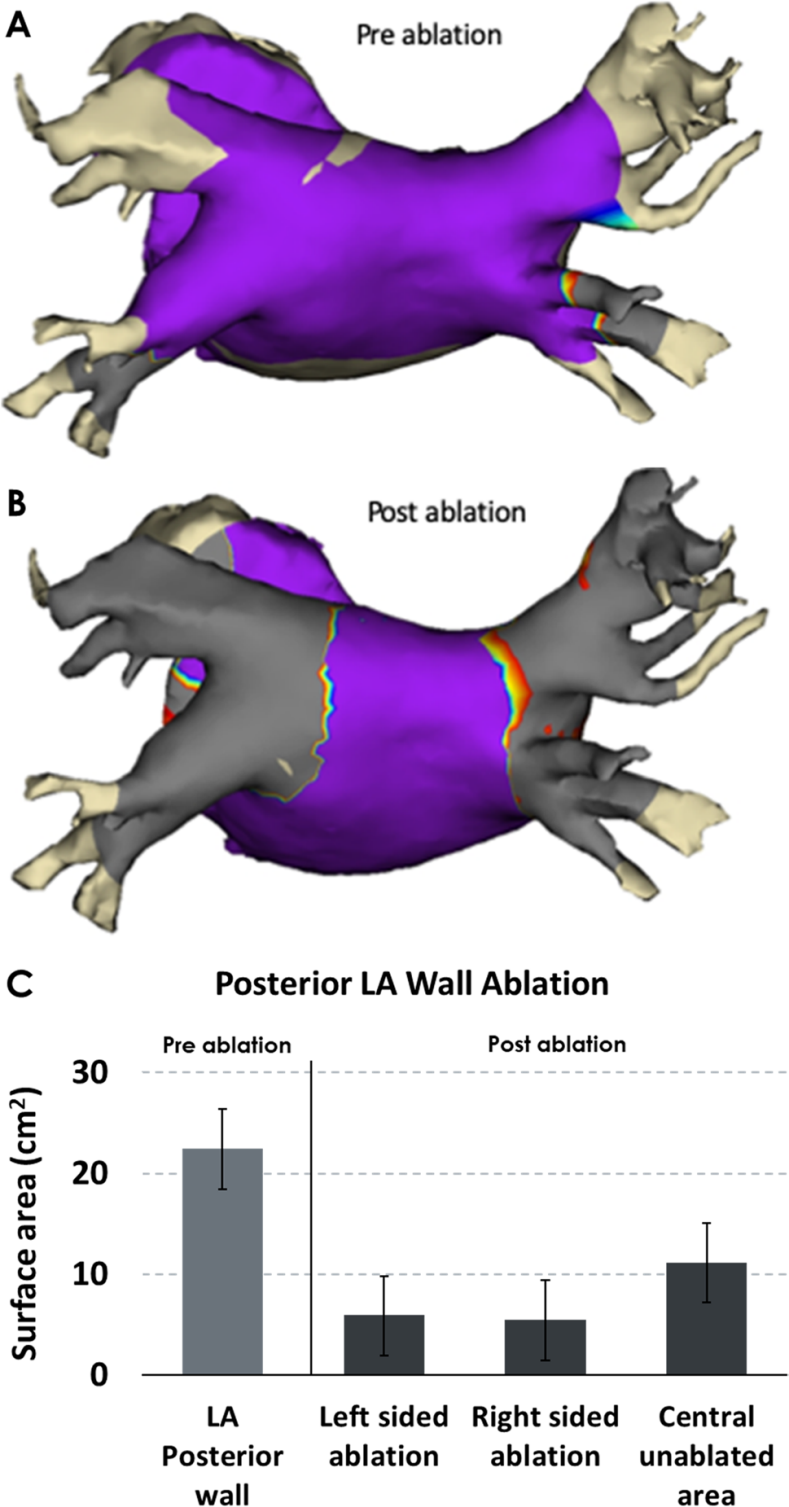
Lesion characterization. Example of the pre- (**A**) and post-ablation (**B**) high-definition electroanatomical maps used to characterize acute lesion formation using the novel cryoballoon. The scale used was 0.2 to 0.5 with purple colour representing voltage > 0.5 mV. (**C)** Pre- and post-ablation mean left atrial surface area of ablation measurements

Acute lesion characterization was completed in all 9 patients enrolled at a single centre. One patient was excluded from the analysis due to insufficient data. Figure 1 shows an example of the pre- and post-ablation high-definition maps used to quantify the extent of ablation on the posterior wall of the LA. Post-ablation mapping revealed that none of the lesions (0/32) was ostial in nature. In this patient subset, the average posterior LA wall area was 22 ± 4 cm^2^ and following PVI, 50% (range: 44–59%) of the posterior LA wall was ablated. No difference was seen between the antral surface area of ablation of the left PVs (5.9 ± 1.6 cm^2^) and the right PVs (5.4 ± 2.1 cm^2^; *p* = 0.63).

### Follow-up data

#### Safety

There were 4 patients (6.9%) with phrenic nerve injury — 3 (4.2%) resolved during the index procedure, with 1 (1.7%) persisting at the completion of the procedure and resolved at the 6-month follow-up. One serious adverse event was reported: acute superficial femoral artery occlusion ipsilateral to the site of venous access occurring 2 weeks following the procedure (1.7%). The patient presented with abrupt onset foot pain. Diagnostic testing with ultrasound and CT identified occlusion of the right superficial femoral artery. At the time of the index procedure, there had been inadvertent puncture of the superficial femoral artery which required prolonged compression of the groin. This was treated surgically with thrombectomy and reconstruction, with successful revascularization. The vascular surgeon found perforation of the right superficial femoral artery, due to the inadvertent puncture of the artery, with associated local thrombosis. The patient fully recovered 6 days after symptom onset.

#### Efficacy

**Fig. 2:**
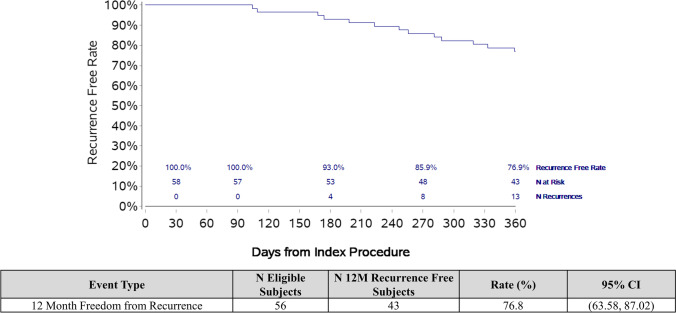
Kaplan–Meier curve of freedom from atrial arrhythmia recurrence (atrial fibrillation, flutter and/or tachycardia)

Complete 12-month follow-up data was available for 56 out of 58 patients. Two patients withdrew from the study prior to the 12-month follow-up. Both were free of atrial arrhythmias at the time of withdrawal but are not included in the final analysis. At 12 months, 43 (77%; *95% CI*: 64–87%) out of 56 patients were free from recurrent, symptomatic atrial arrhythmias (Fig. 2). Of the 13 patients that experienced AF recurrence during the 12-month follow-up, 6 patients underwent repeat AF ablation using standard of care ablation techniques (using an approved RF or cryoablation catheter). All 6 repeat ablation procedures were performed outside of the 90-day blanking period. Comparing those treated with a short tip (*n* = 22) and long tip (*n* = 32) cryoballoon, there was no significant difference between the 12-month recurrence-free rate (86% [*95% CI*: 65–97%] vs. 72% [*95% CI*: 53–86%]; *p* = 0.21) (Table 4). In the single centre subset of 9 patients undergoing acute lesion characterization, 7 out of the 9 patients (78%) remained free of atrial arrhythmia recurrence at 12-month follow-up.

**Table 4:** 12-month recurrence-free rates

	# Eligible	# 12-month recurrence-free	Rate (%)	*95% CI*
All	56	43	76.8	(63.58, 87.02)
Short tip	22*	19	86.36	(65.09, 97.09)
Long tip	32*	23	71.88	(53.25, 86.25)

^*^Two subjects treated with both short and long tip cryoballoon catheters were not included in the short versus long tip sub-analysis

## Discussion

This study evaluates the effectiveness and safety of a novel cryoballoon ablation system, the POLARx Cryoablation System. Patients had paroxysmal AF and ongoing arrhythmia despite the use of class I or III antiarrhythmic medical therapy. Freedom from atrial arrhythmias at 1 year was 77%. Treatment with this novel cryoballoon resulted in phrenic nerve injury in four patients (6.9%); for one (1.7%) patient, this persisted following the procedure but resolved within 6 months.

Numerous technologies have been developed for performing PVI ablation as treatment for paroxysmal AF. The most commonly used of these include point-by-point radiofrequency energy, phased radiofrequency, cryoballoon ablation, and endoscopic laser balloon [2–4]. The effectiveness of these technologies in preventing recurrent atrial arrhythmias at 1 year is approximately 65–83% [2–4]. The freedom of atrial arrhythmias found in this study using the POLARx Cryoablation System is similar to these reports where ablation was used for those patients who had previously failed treatment with either a class I or III antiarrhythmic medication. This freedom from atrial arrhythmias is also similar to that seen in the more recent STOP-AF and EARLY-AF trials, noting the patients enrolled to these trials differed to ours in that they had not previously failed antiarrhythmic medications [16, 17].

Cryoballoon ablation creates encircling PVI ablation and the Arctic Front system is currently the most commonly used single shot technology worldwide. This system has proven to be effective and safe, with procedures of predictable duration [2, 18]. Despite this, the Arctic Front system has several drawbacks. One of these is the increase of balloon pressure and size during the initial portion of the freeze cycle [5], which can disrupt the position of the balloon, thus reducing ablation efficacy. The novel POLARx cryoballoon has been designed to maintain uniform pressure and size throughout the freeze cycle. Because the cryoballoon does not increase in pressure after being inflated, it is more compliant through the start of the ablation. This in turn has raised concerns regarding the location of balloon placement and that ablation may be performed in a more ostial location [6]. Ostial, rather than antral, ablation may result in less effective treatment of atrial fibrillation as electrically active tissue is located within the antrum in addition to the PVs themselves. Lesions at the ostium of PVs also carry safety concerns, resulting in increased risk of PV stenosis and phrenic nerve palsy.

Our data shows that the proximal extent of cryoballoon ablation is antral within the LA, treating 50% of the posterior wall of the LA. This is similar to the extent of ablation that has been described having been created by the Arctic Front cryoballoon system [15]. Such 3D electroanatomical maps accurately identify the proximal extent of ablation but do not identify the distal extent. We have not observed features to suspect excessive distal ablation. The incidence of phrenic nerve injury in this study is similar to others [2, 7–13]. There were no cases of PV stenosis; however, the small size of this study limits our ability to assess for this. In addition, efficacy at 1 year is comparable to that seen with other technologies used for AF ablation [2–4]. This clinical endpoint also suggests that the ablation delivered is isolating PVs and treating antral tissue similar to these other technologies.

One patient experienced a local thrombotic event related to vascular access rather than systemic thromboembolism 2 weeks following their procedure; no patients experienced peri-procedure stroke or transient ischaemic attack. This study did not perform planned MRIs to evaluate asymptomatic cerebral events. The initial reported experience with the POLARx Cryoablation System now totals in excess of 100 patients, and to date ours is the first patient to experience either systemic embolism or stroke [7–13]. Therefore, at this time there does not appear to be a signal for excess thromboembolic complications with this novel system.

There are inherent limitations with a single-arm study design. First, direct comparison between the POLARx Cryoablation System and other ablation technologies, and in particular long-term outcome comparisons with the Arctic Front system, cannot be made. However, there is an existing and emerging evidence base providing a direct comparison at the time of the procedure between these technologies [7–9, 13]. Second, the number of patients enrolled was small, and the centres undertaking these procedures are highly experienced in undertaking catheter ablation for AF with the use of the existing cryoballoon ablation system. Third, these data were part of the first-in-human experience with this technology. This likely limits the ability to generalize the procedure and fluoroscopy times to the real-world setting. Prior studies of novel AF ablation technologies have shown the initial experience to result in both longer procedure and fluoroscopy times than what is subsequently reported in clinical practice with experienced operators [18, 19]. We hypothesize that similar reductions may also occur with this system. To address these limitations, additional, larger studies are currently underway to further determine the efficacy and safety of this novel technology. One such study is the POLAR ICE study, which does not require pre-procedure imaging and does not exclude common pulmonary veins.

## Conclusion

Initial multicentre clinical experience with the novel cryoballoon ablation catheter has demonstrated safety and efficacy of PVI in patients with paroxysmal AF. Ablation with this catheter provides a wide and antral lesion set with significant debulking of the posterior wall of the LA. Further studies are underway to confirm these findings.
